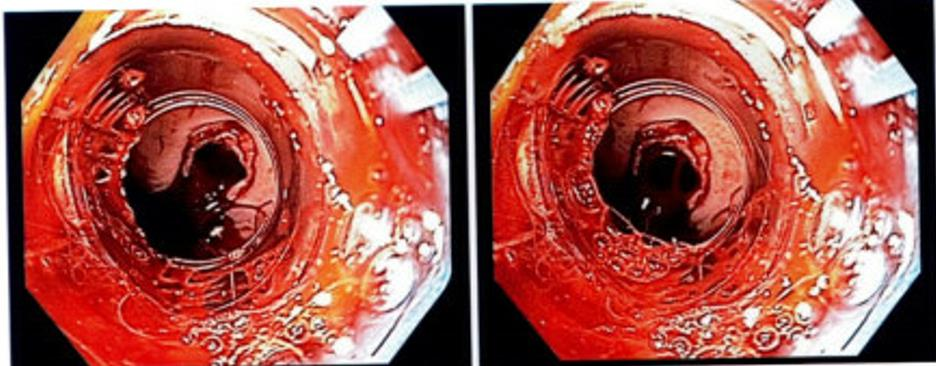# A201 AORTO-ESOPHAGEAL FISTULA AS A COMPLICATION OF ESOPHAGEAL VARICEAL BANDING

**DOI:** 10.1093/jcag/gwad061.201

**Published:** 2024-02-14

**Authors:** U Al Salmi, C Stallwood, K Khan

**Affiliations:** Gastroenterology, McMaster University, Hamilton, ON, Canada; Gastroenterology, McMaster University, Hamilton, ON, Canada; Gastroenterology, McMaster University, Hamilton, ON, Canada

## Abstract

**Aims:**

This is the first reported case of bleeding from an aorto-esophageal fistula as a consequence of ischemic esophageal ulceration post esophageal variceal banding.

**Methods:**

A 46-year-old male presented with epigastric pain for one-week, which was not associated with fever, jaundice, or symptoms of GI bleed. He was hemodynamically stable with an unremarkable exam and basic labs. He underwent a CT scan which showed chronic portal vein thrombosis extending from the main portal vein into the superior mesenteric vein and evidence of portal hypertension, along with massive esophageal varices. Anticoagulation was not started due to risk of GI bleed.

Upper endoscopy confirmed the presence of three columns of large esophageal varices in the distal and mid esophagus, the largest was 3 cm in size. There was no stigmata of recent bleeding and no gastric varices. Seven bands were applied. CT abdomen was repeated on the seventh day of admission, showing extension of portal vein thrombosis. As such, the thrombosis team started a low dose of anticoagulation.

(dalteparin 7500 IU). He tolerated it well and showed no signs of bleeding prior to discharge.

The day after discharge, the patient presented again with an episode of melena. Examination was remarkable for tachycardia, hypotension and melena. Hemoglobin was stable. Patient was admitted with impression of upper GI bleed most likely secondary to post banding ulcer. He was started on medical therapy. Anticoagulation was held. Next day, the patient had an episode of hematemesis with large amount of blood and clots. Emergent upper endoscopy showed active bleeding in the distal esophagus. Further examination showed a full-thickness, 7 mm defect in the lower esophagus with fresh red blood emptying from it into the esophagus (Figure 1). Thoracic surgery was consulted and suspected a perforated esophageal ulceration at the site of previous banding, with an aorto-esophageal fistula. Patient deteriorated immediately and passed away. The cause of death was cardiac arrest secondary to acute blood loss anemia.

**Results:**

AEF is a recognized cause of upper GI bleeding with a high mortality rate. While rare, it can occur secondary to ischemic esophageal ulcer post EV banding.

**Conclusions:**

This is the first reported case of an aorto-esophageal fistula as a consequence esophageal variceal band ligation. While extremely rare, this complication should be considered as a potentially fatal complication of variceal banding, especially in the setting of a diseased or previously repaired aorta.